# A new radio-frequency acoustic method for remote study of liquids

**DOI:** 10.1038/s41598-021-84500-6

**Published:** 2021-03-23

**Authors:** Alexander V. Kramarenko, Andrey V. Kramarenko, Oksana Savenko

**Affiliations:** 1 TREDEX Company Ltd, PO box 11515, Kharkiv, 61001 Ukraine; 2 General and Inorganic Chemistry Department, National Technical University “KhPI”, 2 Kyrpychova Str., Kharkiv, 61002 Ukraine; 3 School of Radiophysics, Biomedical Electronics and Computer Systems, Karazin Kharkiv National University, 4 Svobody Sq., Kharkiv, 61022 Ukraine

**Keywords:** Applied physics, Techniques and instrumentation, Imaging

## Abstract

In the present work, a novel study method of conductive liquids has been proposed. It is based on a discovered phenomenon of radiofrequency anisotropy of electrolyte solution, which arises in response to mechanical excitation of the solution. The phenomenon was observed during the development of a radiofrequency polarimetric contactless cardiograph. The electric field vector rotates after its transition through the pericardial region due to the acceleration changes of blood. Numerous in vitro experiments with monochromatic and impulse acoustic waves always induced the polarization rotation of the RF wave passing through an electrolyte solution. The response obtained from the solutions on acoustic excitation of the Heaviside function form demonstrates the effect of a solution “memory”. The dynamics of this process resembles the spin glasses magnetization. We hypothesized that there was a magnetic moment change within the solution, and the possible reason for it is an appearance of electromagnetic impulse caused by the same acoustic excitation. In a further experiment, we really captured a suspected electrical potential. Given that, we can declare at least three new physical effects never observed before for an electrolyte solution. The study method itself may provide broad options for remote measurement of the electrolyte solution parameters.

## Introduction

Back in 1845, Michael Faraday predicted the electric field vector rotation of linearly polarized light in an applied magnetic field. And When John Kerr found that the refractive index change of optical materials is proportional to the square of the external electric field strength, the wide application of this electro-optic phenomenon began at once. Utilizing a certain substance capable of indirectly changing the polarization of electromagnetic radiation passing through was a necessary condition for Kerr, Pockels (and other) cells to work. In terms of radiophysics, one may represent it like a parametric impact on the system consisting of a transmitter, receiver, and propagated wave. Hence the hypothesis presuming the existence of the Faraday phenomenon at radio frequencies looks plausible enough.

In the present work, we propose a completely new conductive liquids method of study based on a physical phenomenon that has never been noticed earlier. The phenomenon is the radio-frequency anisotropy of electrolyte solution excited mechanically or acoustically. The closest effect we have found in literature is the RF anisotropy discovered for solid ice^1–5^ or liquid crystals^6^, but not for a rather unstructured aqueous solution.

The effect described in the present work was unexpectedly discovered while we were testing our novel device. The device demonstrates a new type of contactless cardiography which is important for the assessment of human health status when the application of ECG electrodes is impossible or inconvenient.

There is a range of papers describing the contactless ECG registration devices developed in recent years. Nowadays there is a trend of replacement of a high-impedance low-frequency amplifying technique^7–11^ with an active S- or C-band radar systems^12–16^ which are directly measure the displacement of the cardiac wall instead of electric potential recording. And they really seems to be more promising due to their better signal/noise ratio within a working band.

Our contactless polarimetric cardiography system was developed primarily for car drivers. In such conditions, one has to adjust for the interference because of vibration and sensor displacement. However, up-to-date monostatic Doppler radars require either on-body antenna fixation^12^ or additional information about the variation of their relative position, which is several orders of magnitude greater than the amplitude of the periodic baseband signal caused by heart and breathing activity^13^. To obtain a good reflected signal (in the case of monostatic radar) the required radiation power will always be much higher than in the case of “open” operation when the locator is bistatic^17^. Minimizing the power consumption during prolonged operation of the device is one of the most important equipment requirements^18–20^. To make the total RF energy flux density be lower than $25~\upmu \mathrm {W/cm^2}$ to comply^18^ we have used a very low power transmitter ($-6 ... -\!\!12~\text {dBm}$). Moreover, according to our measurements, when the wave from our device passes through the human body, attenuation is small, no more than 20 dB. It means the negligible radiation powers of $-40 ... -\!60~\text {dBm}$ can be easily achieved and then the total absorbed dose of RF energy will be reduced. The signal-to-noise ratio remains good precisely because of polarimetry since an external non-polarized interference is the same in both receiving channels and is subtracted mutually.

The polarimetric method also does not impose high requirements on the spectral purity of the local oscillator signal, which is the main fundamental sensitivity limitation of continuous-wave Doppler radars^21^. Therefore, the gain in minimizing the power of the emitted signal also increases many times with respect to them.

## Method basics

### A brief description of the device

**Figure 1:**
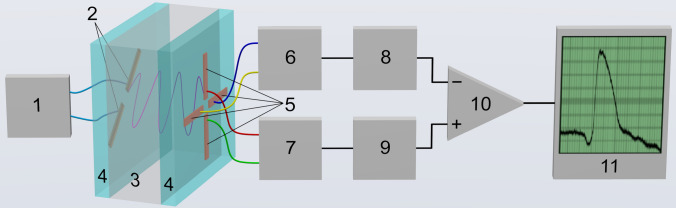
A simplified block diagram of the device for recording of RF wave polarization direction deviation. 1—a continuous wave transmitter 433.82 MHz, − 12 dBm; 2—a transmitting antenna; 3—an object under study; 4—a dielectric material; 5—orthogonal orientation quadrature antennas; 6—a channel X RF amplifier; 7—a channel Y RF amplifier; 8—X channel amplitude logarithmic detector; 9—Y channel amplitude logarithmic detector; 10—an instrumentation amplifier; 11—a registering device (X axis is time, Y axis is deviation). The antennas are shown as Hertz’s half-wave dipoles, it is clear that their real configuration is going to be different. Transmitting antenna is deviated at angle of ${45}^{\circ }$ relative to both receiving ones.

Figure 1 shows a signal pathway block diagram. RF signal of the transmitter *1* excites the transmitting antenna *2*. A linearly polarized wave passes through the object *3* (some abstract medium is shown here) to be received then by two orthogonally oriented antennas *5*. The input stages *6* (*X* channel) and *7* (*Y* channel) amplify the corresponding signals that pass to the amplitude logarithmic detectors *8* and *9* next. The differential amplifier *10* provides an output signal proportional to the difference in logarithms of amplitudes of low-frequency signals. The standard $-3$ dB bandwidth (with 0.3 Hz and 75 Hz cutoff frequencies) is applied. The dynamics of the process can be observed via the oscilloscope *11*. So, the multistatic radar emitting a continuous monochromatic wave passing through the pericardial region has been implemented. Such registration principle makes our device much safer for long-term recordings (monitoring) in the sense of absorbed dose of radiation. A comprehensive paper about our system is going to be printed in the offing.

### Basic relationships between the signals

Of course, we are far from claiming our transmitting antenna emits exactly the linearly polarized wave having no traces of elliptical polarization. Nevertheless, we can reasonably assume the imaginary component of the Jones vector to be small in comparison with the real one^22–24^. It suggests the relation of received RF-signals is proportional to the tangent of the shift between transmitting and receiving antenna until there is no anisotropic medium between the antennas. To provide this, we have adjusted the antennas’ disposal so that the logarithmic detectors’ positive output signals were out of phase relative to a chosen level. Considering this level as zero one, we can write the final relations as:

1 $$\begin{aligned} \left\{ \begin{array}{l} \log U_y + \log U_x = 0, \\ \log U_y - \log U_x = \log \frac{U_y}{U_x} = \log (\tan \alpha ) = U_{\mathrm {out}}/k\\ \end{array} \right. \end{aligned}$$

where $\alpha$ is the shift, $U_{\mathrm {out}}$ is output voltage and *k* is the gain of op-amp *10*. As the shift is ${45}^{\circ }$ (see Fig. 1), the relation of the amplitudes is equal to one, so the relation logarithm is zero. We have also performed some gain calibration deviating $\alpha$ by $\approx \pm {30}^{\circ }$ from the ${45}^{\circ }$ level and adjusting $U_{\mathrm {out}}$ to be inside $\pm 1~V$ range. As a result, we have derived a calibration dependence of the form of:

$$\begin{aligned} \alpha \approx \arctan (\exp (1.32U_{\mathrm {out}})). \end{aligned}$$

It has turned out to be linear enough within $U_{\mathrm {out}}$ range of $\pm 0.2~V$; the Taylor series in the vicinity of the point $U_{\mathrm {out}}=0$ limited to the first three significant terms is

2 $$\begin{aligned} \alpha \approx 45+37.8U_{\mathrm {out}}-11.0U_{\mathrm {out}}^3. \end{aligned}$$

The appearance of an anisotropic medium between the antennas changes the output signal and, hence, the $\alpha$ angle variation is observed.

**Figure 2:**
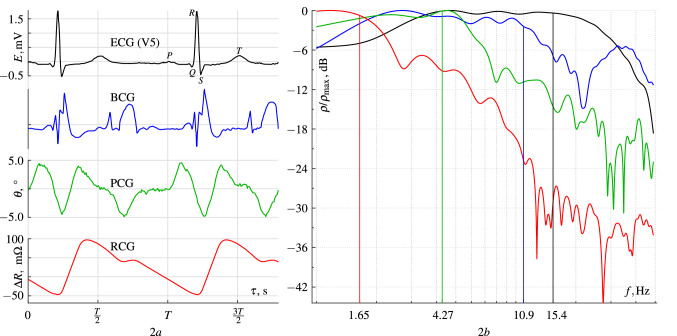
Graph 2*a* shows the polarimetric cardiogram (PCG, green) we have recorded synchronously with a standard ECG in V5 lead (black) and rheocardiogram (RCG, red) combined with the ballistocardiogram^25^ (BCG, blue). Graph 2*b* shows their normalized power spectra and corresponding median frequencies. The heartbeat period $T\approx 0.78$ s.

A propagation medium is, commonly, described^26^ by $2\times 2$ scattering matrix, or operator, acting on the Jones vector of the wave. Generally, such an operator may change the polarization ellipticity. But we assume the pericardial region to possess only the property to rotate the polarization direction at a certain angle, making it possible to write the corresponding rotation operator $\Vert {R(\theta )}\Vert$ as the following^26^:

$$  \overrightarrow {E} _{0}  = \left\| {R({\theta })} \right\|\overrightarrow {E}  = \frac{1}{{\sqrt 2 }}\left\| {\begin{array}{*{20}l}    {\cos{\theta } - \sin{\theta }} \hfill & {\cos{\theta } + \sin{\theta }} \hfill  \\    { - \cos{\theta } - \sin{\theta }} \hfill & {\cos{\theta } - \sin{\theta }} \hfill  \\   \end{array} } \right\|\left\| {\begin{array}{*{20}l}    {E_{x} } \hfill  \\    {E_{y} } \hfill  \\   \end{array} } \right\|, $$

where $E_0$ It is a Jones vector of received wave in the receiver antenna basis; It is a Jones vector of the transmitted wave in the transmitter antenna basis; $\theta = \alpha -{45}^{\circ }$ is the angle of polarization direction rotation conditioned by the presence of an anisotropic medium. All our further considerations are related to this parameter. There were no $\theta$ values more than $\pm {30}^{\circ }$ recorded during testing, so all of $\alpha$ values are in the first quadrant. It allows us not to take into account the phase relation of the RF-signals received. Indeed we are not able to distinguish polarization vector rotation and polarization ellipticity changing on the described device, observing only the output signal of the op-amp *10*.

Thus, we can record (with acceptable measurement error) UHF-band wave polarization change because of propagation medium anisotropy, disregarding the slight ellipticity of the wave. We have chosen the frequency (433.82 MHz) so that the half-wavelength (34.5 cm) approximates the linear dimensions of the heart and large vessels.

## Method testing

### Treatment of the data obtained and a new hypothesis

A polarimetric cardiogram (PCG) had been expected to have minimum artifacts and was recorded by the contactless method (see the video^27^ of the first successful attempt of such recording). The PCG was recorded from a healthy patient placed in the middle between the receiving and transmitting antennas at about 1 m distance from each other.

The left graph in Fig. 2 shows an electrocardiogram (ECG), a PCG, and a rheocardiogram (RCG) we have recorded synchronously from the same patient. We also have added another patient ballistocardiogram^25^ (BCG) adjusting its heartbeat period to correspond to our record. The right graph shows the corresponding power spectra of a heartbeat period. Hann window (Tukey window^28^ having $\alpha =1$) has been applied to data prior to Fourier transform.

At the beginning of the polarimetric cardiography investigation, we assumed the polarization direction rotation mechanism be caused by the heterogeneity of a heart as a propagation medium. It is known that cardiac muscle (myocardium) and large vessels connected to it are deformed and rotated during the systole. This is reliably sustained with dynamic studies of cardiac activity^29^. Indeed, the radio wave passing through the pericardial region changes its polarization direction because of the heterogeneous tissues shifting and rotating. It means that the expected rotation of polarization direction might be synchronized with “external” cardiac ballistics.

But the PCG pulses (see Fig. 2) mismatch the initial hypothesis. They are more prolonged than ECG pulses, and start earlier, and cease later, what defied explanation. PCG signal is in good accordance with intracardiac (NB !) hemodynamics. It has turned out that $\theta$ change is associated rather with “internal” cardiac ballistics than with “external” one. In other words, the rotation is caused by the moving of intracardiac and intravascular blood (inside the heart and large vessels connected to it).

We hypothesized that radio-wave polarization direction after its passage through the pericardial region depends on the electric properties of the medium itself, i.e. on the properties of the blood the radio wave penetrates. To prove this hypothesis, we calculated the median frequency for each of the spectra we had obtained. The calculated values are shown as the *x*-axis tick labels of the right graph in Fig. 2.

It is well known that ECG represents the propagation process of an electric excitation wave through the myocardium. Such a process might be considered as virtually inertialess, and its spectrum possesses a maximum median frequency in comparison with any other cardiac monitoring methods. A ballistic cardiogram (BCG) presents the mechanical oscillations initiated by an electric signal. Since the heart contraction energy remains constant, the BCG spectrum should demonstrate a redshift relative to the ECG spectrum due to the inertia of the masses involved in the oscillation process. The spectra graph (Fig. 2) shows it well ($10.9~\mathrm {Hz}<15.4~\mathrm {Hz}$) despite the high frequencies presence caused by the heart valves work^30,31^. If the PCG waves corresponded to the blood acceleration/deceleration and not to the myocardium contraction, the spectrum of the PCG would contain far lower frequencies than the BCG spectrum. It really takes place as we can see ($4.27~\mathrm {Hz}<10.9~\mathrm {Hz}$) what backs our hypothesis. And, of course, the RCG spectrum should have the lowest median frequency since a rheocardiogram signal depends (according to Kedrov^32^ and Nyboer^33^) on a tissue volume variation affected by newly run blood. The damping effect because of the mass involved in the process is maximal in this case, hence the corresponding spectrum possesses the lowest median frequency ($1.65~\mathrm{Hz}$).

The left diagram in Fig. 2 also shows that the beginning of the $\theta$ deviation coincides with the peak of the P wave on the ECG. The P wave corresponds to the beginning of an intracardiac discharge of blood into the ventricles when cardiac muscle barely starts to contract, but blood is still almost motionless. This coincidence allows us to assume that the immobile (or moving steadily) blood may rotate the radio wave polarization direction by a (zero or non-zero) constant angle (not changing in time). But the variations of the rotation angle could be associated only with blood acceleration or deceleration, i.e. a nonstationary blood motion. Moreover, a non-zero PCG signal was registered even after the cardiac contraction had finished. It was consistent with the reverse blood flow within the aorta and pulmonary artery, which closes the heart valves when a rapid bloodstream slowdown occurs.

Thus, we hypothesize that the polarization direction rotation is caused not only by heart displacement and rotation but rather by the moving blood properties.

### Reproducing the phenomenon *in vitro*

#### A simple setup

**Figure 3:**
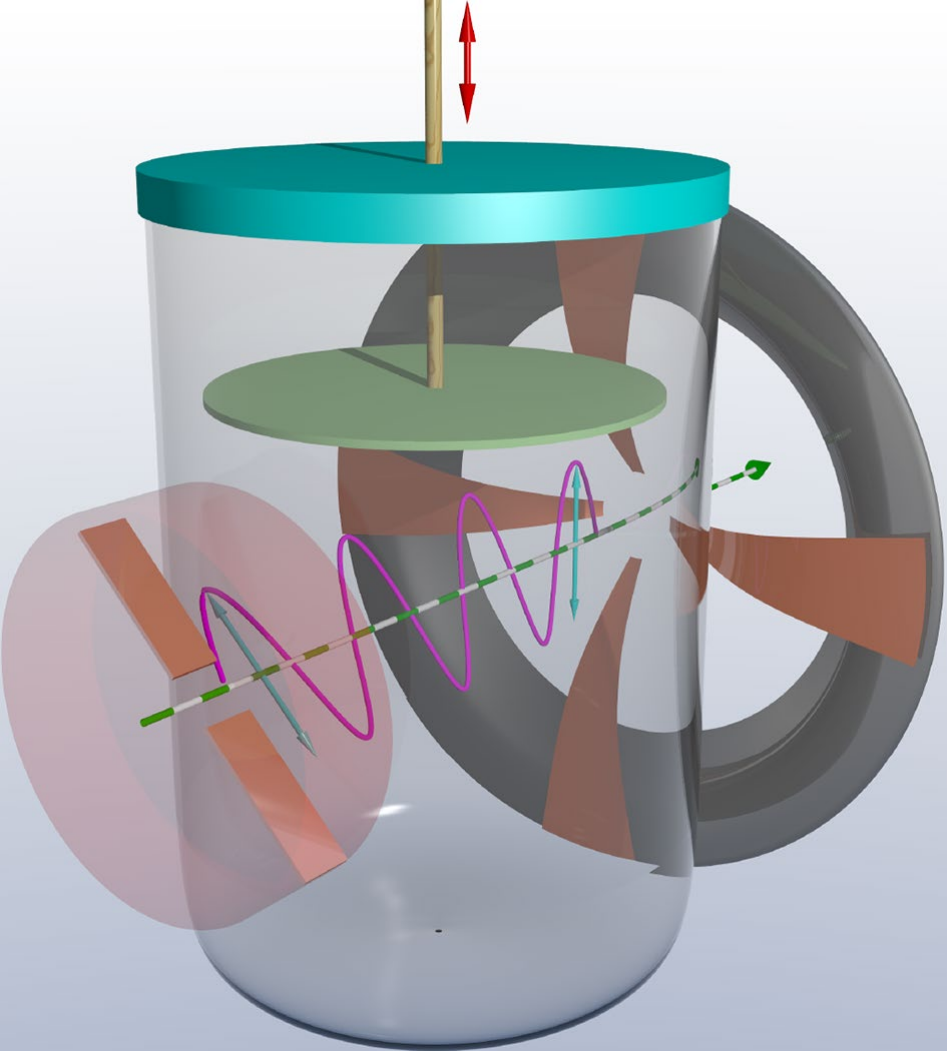
The experimental setup scheme. A shortened transmitting antenna (surrounded by a radar-absorbing case on the sides) is on the left, while a receiving slot antenna with QAM is on the right. The shift between the antennas polarization is ${45}^{\circ }$.

In order to examine experimentally our hypothesis about the dependence of polarization direction rotation on the blood (conductive medium) movement, we assembled the setup illustrated by Fig. 3.

There is a covered container between the transmitting and receiving antennas. A thin polystyrene disk mounted on a long dielectric pusher is placed inside the container. The actuating pusher applies an external force to the disk and makes it move. The container is filled with an electrolyte solution without any air residues. Such setup implies no sway or shape change of the solution so long the disk moving. Thereby the distortion of the scattering field affecting the measurements is observed or, simply put, mechanical excitation. The disk was displaced in an axial direction by the pusher because of the weight change after the recording had started. The weight drop was provided either by a soft push through the shock-absorbing pad or by a sharp blow. The experiment was carried out at an ambient temperature of $+\,20^{\circ }\hbox {C}$.

The absence of any response has been checked previously on the disk moving inside the empty container and one filled with distilled water. In the first case, there was no signal at all. In the second, a barely noticeable signal was recorded at the noise level. The response signal disappeared completely when distilled water was replaced with a bidistilled one.

Then we have held a series of measurements on different solutions. The first experiment was carried out on distilled water and citric acid dissolving slowly within it. The experiment on distilled water and citric acid was the very first approximation of a cardiac system environment. Here we saw the response increased when the acid dissolved.

Further, we have investigated the same process on NaCl and KCl solutions having approximately equal molar concentrations. The same equipment as for the cardiogram recording was used.

It should be noted that these experiments are easy to implement and are highly repeatable. Despite this, the effect has remained unnoticed since the invention of radio communications.

#### A mechanical excitation in simple setup

**Figure 4:**
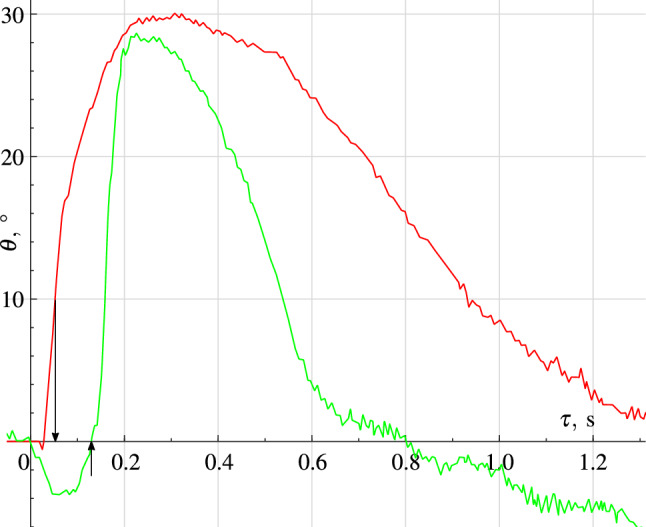
The $\theta$ angle variation during a soft push of the disk (green curve). In the experiment on a tinted solution, it was seen that the jets flowing around the disk move in the opposite direction at a higher speed than the disk itself. A red curve refers to the same but in response to a sharp hit on the pusher. The jet flow differs from the previous observation. For both curves, the external force starts to act at zero time and ends at the time marked with the arrow.

When the container was filled with an isotonic NaCl solution, any displacement of the disk always caused an appearance of a high-amplitude signal at the detector’s output. The amplitude of the output signal enhanced with the pusher acceleration increase. Figure 4 shows the waveforms of the process. We did not find detectable differences in the response for NaCl and KCl solutions having approximately the same molar concentrations of $\approx 0.157$ mol/l.

The experiment on more concentrated NaCl solution confirmed the growth of the response with the growth of concentration ceteris paribus. The response initially passed through the peak of intensity and decreased as the concentration approached the saturation, and then the response was hardly observed within the saturated solution. We can assume that the described effect is recorded by our equipment only for sufficiently concentrated but unsaturated solutions. We did not find detectable differences in the response for NaCl and KCl solutions having approximately the same molar concentrations. No experiments with frequency variation were carried out.

**Figure 5:**
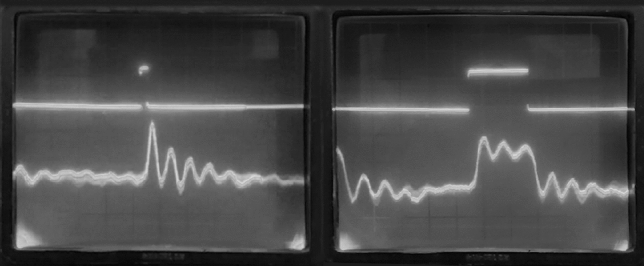
Left waveforms show a system response on the repetitive acoustic pulse 1 ms long. Waveforms on the right correspond both to the pulse duration and repetition frequency increased. The damped oscillatory process is clearly visible, possibly because of an echo in the container. The upper beam shows a hydroacoustic radiator input signal, while the lower one is the op-amp output voltage. The electric power of the impulse applied to the hydroacoustic radiator is equal to 1 mJ. The polarization direction of the wave is deviated from the soundwave propagation vector by ${45}^{\circ }$. We were able to observe the effect confidently down to pulse energies of $1~\upmu \mathrm {J}$.

**Figure 6:**
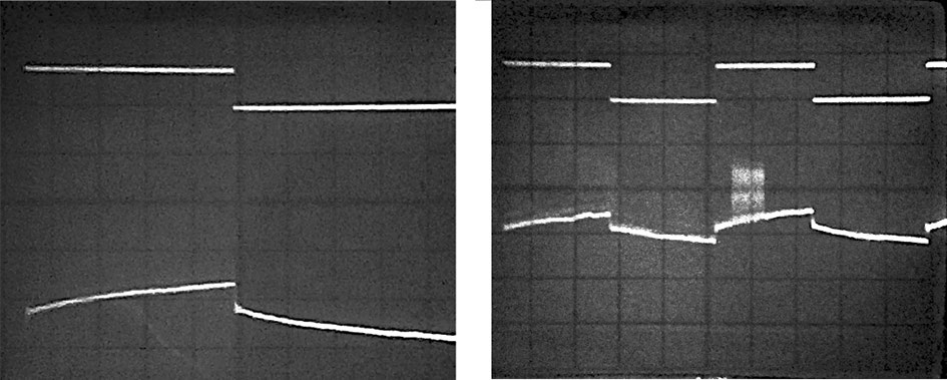
A system response on the Heaviside function signal (upper beam) is shown on the left. Recording duration is 2.5 s. The equipment bandwidth starts at zero frequency. It is easy to observe that after a rapid, momentary $\theta$ deviation because of the acoustic wave propagation, the further post-impulse rotation continues in the same direction. There is a 5 s fragment of the record on the right, which shows an unsuccessful attempt to interfere to the post-impulse rotation process by turning on (briefly) an additional more powerful transmitter. The interference is visible on the lower beam of the oscilloscope.

#### An acoustical excitation in simple setup

However, in order to find a supposed acoustic phenomenon we have held one more experiment under rather different conditionals. We recorded the response from the solution excited with an immersed into it hydroacoustic radiator. Figures 5 and 6 show the corresponding waveforms recorded from the operational amplifier output.

Completely unexpected behavior of the studied system was revealed in the experiment with a hydroacoustic radiator. A reaction of electrolyte solution to the acoustic wavefront passage was very prolonged contrary to our expectations. Thus, a long-term (in comparison with typical solvent relaxation times) “memory” was detected for the system.

As one may remember, we observed polarization direction rotation within the heart medium conforming with the ECG signal earlier in Sect. 3.1. But here, we had a slow $\theta$ variation continuing after its rapid deviation at the moment of the acoustic wave passage. Thereby the anisotropy degree of the entire volume increased (see Fig. 6) within tens of seconds after the pulse. The $\theta$ angle value passed through the maximum and went then back to its initial value (before the pulse) after 30 seconds or even more.

This makes us conclude: when the wavefront passes through the medium, anisotropy arises almost inertialess, i.e. the times observed were less than $50~\upmu$s at the present installation (and they are most likely much less than this value). Anisotropy persists for at least tens of seconds after the wavefront transit. It increases with time (see Figs. 5, 6), then reaches its maximum, decreases, and disappears. The passage of the acoustic wave reverse front changes the sign of the arisen anisotropy (see Fig. 6).

To exclude the influence of RF energy “pumping” into the studied object, we have attempted to interfere in the process with another more powerful ($+$ 20 dB) than the initially used transmitter and had the same frequency (but different polarization direction). It was turned on for a very short time but had no effect on the process of post-impulse $\theta$ variation (see Fig. 6). It is obvious that we can neglect the “observer effect”, i.e. there are no changes in the structure of the observed object because of the radio signal passing through it.

Any manipulations with the power and/or modulation of the radio signal do not affect the time dependence of anisotropy (see Fig. 6).

Our experiments cannot yet provide a quantitative estimation of the phenomenon registered that is outwardly similar to the effects discovered by Kerr and Pokelson. It is not mentioned in the main physical reference books (for example,^34^). However, we have experimentally established now that the unsteady motion of the electrolyte solution affects the polarization direction of the RF wave passing through it.

#### Further investigation of the processes

**Figure 7:**
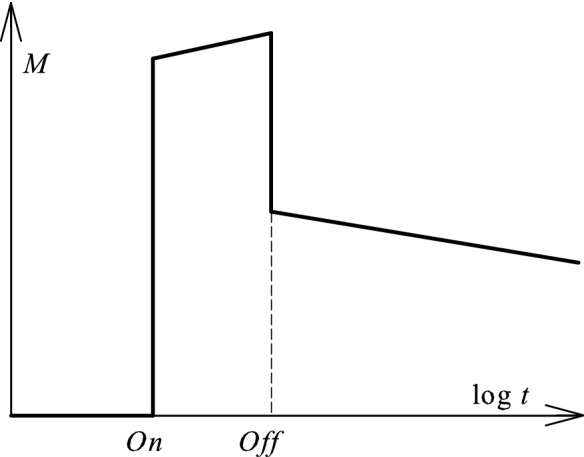
Magnetization *M* of spin glass as a function of time after turning on and off the magnetic field.

At this point, we have fixed two unexpected effects within an electrolyte solution. Regarding the memory phenomenon, we found its dynamics (and giant relaxation times) looking similar to the process of spin glass magnetization shown at Fig. 7^35^.

**Figure 8:**
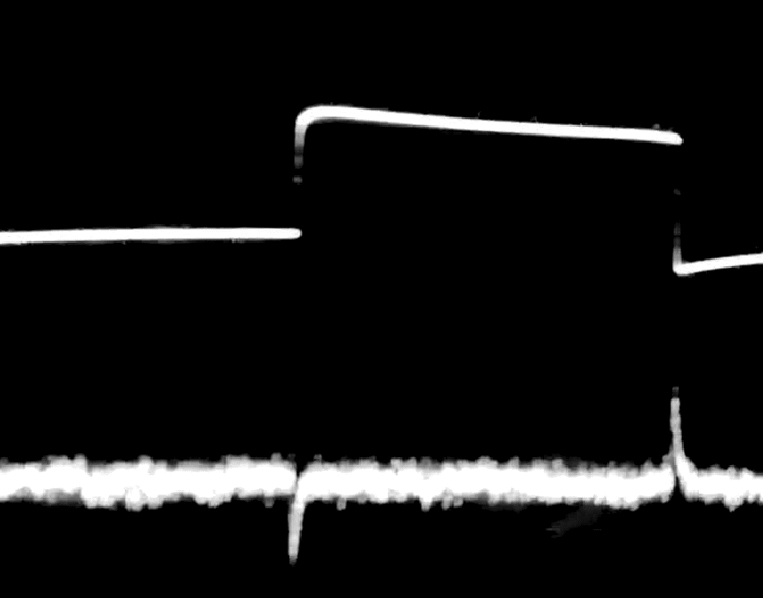
The upper beam is an acoustic signal in a 2 L container. The lower one is an electrical signal, which appears in the solution as the acoustic wave passes, recorded by silver chloride electrodes. The polarity of the electrical signal depends on the orientation of the electrodes relative to the acoustic radiator.

Our further investigation in this direction led us to searches for the mechanism of the suspected magnetic moment change within the solution. As the derivative of the system response (see Fig. 6) to the Heaviside function in our case is not merely delta function but has an exponentially decreasing right tail, we looked for the process which could match such a view and could also be responsible for the magnetic moments change. The only process capable of this change occurrence is the appearance of an electromagnetic pulse. Thus our next experiment became an attempted to capture its presence. And indeed we have registered the uprise of the electrical potential (see Fig. 8) in the solution due to acoustic excitation (Sic!).

**Figure 9:**
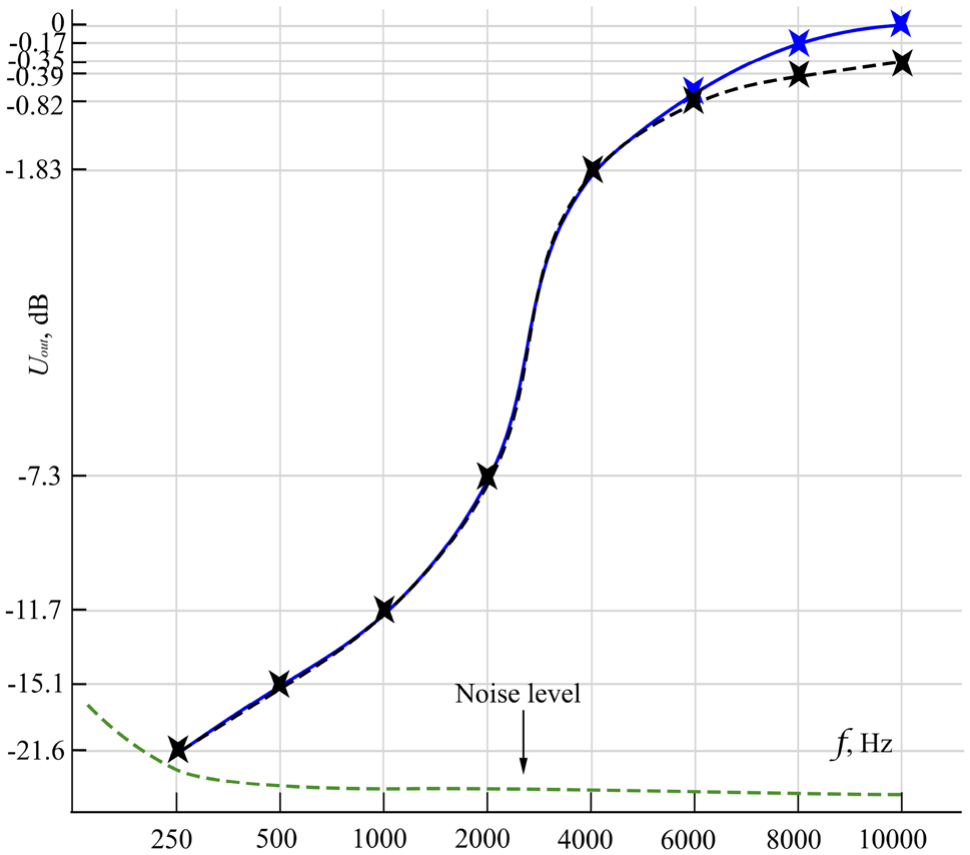
Output signal dependence on acoustic excitation frequency, or shortly the FRF of the system. The blue solid line is FRF at $0^{\circ }\hbox {C}$, and the black dashed line is FRF at $+\,20^{\circ }\hbox {C}$. 0 dB response is observed at 10 kHz and $0^{\circ }\hbox {C}$.

The validity of our assumption can be established in light of the measured output signal dependence on acoustic excitation frequency illustrated in Fig. 9. The form of a signal in the time domain has to correspond with its view in the frequency domain, i.e. energy spectrum. It is also obvious that an FRF at zero frequency for our electric impulse must be zero and then increase in the blue region of the spectrum. That’s right what we see.

### Ethical statements

The given experiments are conducted in accordance to Declaration of Helsinki (2000), Constitution of Ukraine (article 45) and Order of the Ministry of Health of Ukraine No. 281 dated 01.11.2000, as the research was held in Ukraine and these documents are a ground for any biomedical research within the country. Besides, informed consent was obtained from all participants of the study.

## Discussion

A classical Debye IVP appearance mechanism suggests the hydrodynamic forces drive cations and anions differently in a moving liquid. It may occur due to non-equal masses of ions, their different radii, and hydration numbers. Hence the displacement of negative charges in relation to positive arises in accordance with the theory.

The opposite charges displacement, in turn, should cause the occurrence of parallel oriented water molecules statistic dipole structures in the solution. Then the polarization direction of the radio signal passed through it rotates as the orthogonally polarized waves generated by the antenna have different speeds in an anisotropic medium. In accordance with Debye theory modified in the recordings of the acoustically induced EMF show that its value is proportional to the derivative of the acoustic signal which excites the conductive medium (see Fig. 8).

The intensity of response from KCl solution indicates the hydrodynamic explanation is at least insufficient. If our proposed hydrodynamic explanation were close to reality, then (ceteris paribus) the intensity would have been much less than one from NaCl solution because the dielectric friction coefficients for $\hbox {K}^{+}$ and $\hbox {Cl}^{-}$ in water are very close to each other at the range of temperatures from $+\,5^{\circ }\hbox {C}$ up to $+\,25^{\circ }\hbox {C}$^36,37^, but it has been proven experimentally that the IVP responses for NaCl and KCl are very close to each other ceteris paribus. This fact backs our experimental results showing an equal response from both the solutions. It is also the reason why we have to consider our newly described effect and Debye IVP as the strongly interconnected phenomena. Withal, the existing theory predicts an increase in IVP with reciprocal frequency in contrast to our experimental results (see Fig. 9). It is very likely that the theory does not apply to the case of low frequencies and high concentrations due to the assumptions made during the derivation of the main equation. One may find useful some novel theoretical approaches capable of the unsteady fluid flow description at the microscopic level, for example, the so-called theories of complex fluids^38^.

It is very likely that a simple hydrodynamic description of the observed effect, even within the framework of a more perfect model, cannot correctly explain the phenomenon of residual anisotropy, the so-called “memory”. Normally it should not have occurred.

Taking into account the thermal motion in the solution, one could hardly assume the presence of any molecular vibrational processes that cause such a long “memory”—they should have been leveled out by thermal noise rather quickly. Moreover, the total energy of the thermal motion of water molecules is immeasurably greater than the energy introduced into the system by an acoustic pulse. The energy of a pulse of $50~\upmu$s duration emitted by a 1 W hydroacoustic radiator was of the order of $50~\upmu$J. The front of such a wave, according to our data, rotates the polarization direction by 10$^{\circ }$approximately.

Let’s assume the anisotropy to be caused precisely by the orientation of the water molecules dipole moments. Then, the integral hodograph of the molecular motion, which is a sphere in an isotropic medium, should take the form of an ellipsoid with any significant difference between the major and minor semiaxes. One should take into account that the enthalpy of water at $+$ 20$^{\circ }$C is 84 kJ/kg relative to the triple point and the mass of the solution taken for our experiment was about 2 kg. Thereby, it is hard to anticipate the acoustic pulse of the utilized hydroacoustic radiator to be capable of introducing sufficient energy in accordance with our assumption. The estimates of the energies of the acoustic pulse and the thermal motion of water differ by approximately 9 orders of magnitude.

Also, some hope was given by the known experimental fact that two spin isomers of water can be separated under very specific conditions^39^. In this case, the time that equilibrium between water isomers takes to establish coincides (in order of magnitude) with the relaxation times we observe.

The likely physical process that remained suitable for explaining the observed giant relaxation times was the interaction of the nuclear spin angular momentums of hydrogen atoms of water molecules^40^. One might use the properties of spin glass as a prototype and then refer to the behavior of the solution under the study to the Edwards-Anderson model^41^. Id est, there is no random directions of spin, but a degeneracy in various spin configurations within the medium of acoustic wave propagation. Withal the number of possible metastable states is infinitely large, and they possess a very long relaxation time. The authors cannot yet unambiguously correlate these states with the existing physical objects in the electrolyte solution (hydrated ions, individual water molecules, or their clusters) to completely unravel the problem.

Meanwhile, our latest hypothesis (according to the theory accepted for the spin glass) is the presence of frozen magnetic moments within the solution. From this, the influence of an acoustic wave should cause the appearance of an electromagnetic pulse in the solution. We obtained the evidence of this event occurred but, yet we’re not able to indicate precisely what physical mechanism is behind. The electric potential appearance may be considered as described in^42^. It states the difference of electric potential between two points of an electrolyte solution, which is moving with alternating acceleration. The mechanism of such potential appearing was considered as a shift of the opposite charges existing in a plasma-like electrolyte, analogously to shift, which causes the Langmuir waves in real plasma. However, one cannot exclude the possibility that this potential arises, for instance, due to the acoustic modulation of the Grotthuss effect.

Of course, an exhaustive theoretical justification is required here and the only thing we can warrant is the results of our experiments.

## Conclusions

Summarizing our experiments, we can highlight the following.

1. An acoustic or mechanical excitation of pure water does not cause any discernible rotation of RF signal polarization. Keeping the constant level of acoustic excitation, we have revealed that the $\theta$ rotation angle grows with increasing concentration of electrolyte in water. The response decreased when NaCl solution started to slightly approach the saturation and, for the saturated solution, it practically ceased on our equipment.
2. An experiment with KCl solution having a molar concentration of 0.157 mol/l, which is close to that for an isotonic NaCl solution, did not reveal an experimentally detectable difference in response between them.
3. The magnitude of the resulting RF anisotropy depends on the energy of the front of the acoustic wave passing through the electrolyte solution and does not depend on the power of the RF transmitter.
4. When the wavefront passes through, anisotropy arises almost inertialess, i.e. the times observed were less than $50~\upmu$s at the existing installation (and they are most likely much less than this value).
5. Anisotropy persists for at least tens of seconds after the wavefront passing. It even increases with time (see Figs. 5, 6), then reaches a maximum, decreases, and disappears. This turned out to be completely unexpected. Any manipulations with the power and/or modulation of the radio signal do not affect the time dependence of anisotropy (see Fig. 6). Thus, a peculiar effect of “memory” is registered, which requires a consistent explanation.
6. The passage of the reverse front of the acoustic wave changes the sign of the anisotropy arisen (see Fig. 6).
7. During the passage of an acoustic wave through an aqueous electrolyte solution, we registered a low-level electric potential, the so-called IVP (see Fig. 8), probably, responsible for the memory effect.

And finally, we would like to share our view on the prospects of the observed phenomenon.

1. A completely non-contact method may be promising for industrial applications, e.g. for monitoring liquids moving through the pipelines (see our demo-video^43^), detecting unacceptable vibrations, turbulences, etc. We suppose that the method is applicable for fundamental physics research as well as for various branches of industry, biology, and medicine, oceanography, and geology.
2. The sensitivity and selectivity of sonar stations may be increased due to the use of a polarimetric sensor as a hydrophone. Currently, the phase transition of sound waves passing through the propagation medium to a sensitive element is eliminated in all existing microphones.
3. Measurement of the angle of RF polarization direction after the passage of a single short acoustic pulse through the body tissues allows us to develop a fundamentally new method of single-pulse ultrasound introscopy.
4. In our opinion, the effect can be widely used in biophysics and medicine, taking into consideration the necessity for non-contact research methods. Monitoring of operator’s vitals is the case, e.g. the car driver’s heart rate (see our demo-video^44^) and the integral assessment of the heart pumping function (see our explanation video^27^).
5. The practical application of the effect detected is already possible using the developed equipment and signal processing algorithms since the proposed sensor will perform the functions of a “finite state machine” (i.e. time-invariant transcoding). The reference data in read-only memory is enough for it to work.
6. We believe that the theoretical substantiation of the observed phenomenon requires wide cooperation of specialists in various fields.

## Data Availability

No datasets were generated or analysed during the current study.
