# Publisher Correction: A new radio-frequency acoustic method for remote study of liquids

**DOI:** 10.1038/s41598-021-92867-9

**Published:** 2021-06-22

**Authors:** Alexander V. Kramarenko, Andrey V. Kramarenko, Oksana Savenko

**Affiliations:** 1 TREDEX Company Ltd, PO box 11515, Kharkiv, 61001 Ukraine; 2 General and Inorganic Chemistry Department, National Technical University “KhPI”, 2 Kyrpychova Str., Kharkiv, 61002 Ukraine; 3 School of Radiophysics, Biomedical Electronics and Computer Systems, Karazin Kharkiv National University, 4 Svobody Sq., Kharkiv, 61022 Ukraine

Correction to: *Scientific Reports* 10.1038/s41598-021-84500-6, published online 23 March 2021

The original version of this Article contained errors in References 27, 43 and 44, where hyperlinks to external data were omitted and as a result were incorrectly given as:

27. Kramarenko, A. V. Demo of non-contac polatimetric cardiograph testing, ( accessed: Jul 23, 2020a)

43. Kramarenko, A. V. Demo of an industrial application (pump work control) of a non-contact rf registration device (accessed: Nov 3, 2020”b).

44. Kramarenko, A. V. Demo of a car driver monitoring system (accessed: Oct 13, 2020c).

The correct references are listed below:

27. Kramarenko, A. V. Demo of non-contac polatimetric cardiograph testing, https://www.youtube.com/watch?v=gOGvGjJ2QnI (Accessed 23 Jul 2020a).

43. Kramarenko, A. V. Demo of an industrial application (pump work control) of a non-contact rf registration device, https://www.youtube.com/watch?v=z-1pzf3iUyM (Accessed 3 Nov 2020b).

44. Kramarenko, A. V. Demo of a car driver monitoring system, https://www.youtube.com/watch?v=yB9uKd3MNxU (Accessed 13 Oct 2020c).

The original Article has been corrected.

